# Supplementary material for: Honey bees and bumble bees occupying the same landscape have distinct gut microbiomes and amplicon sequence variant-level responses to infections
Source: PeerJ. 2023 Jun 9;11:e15501. doi: 10.7717/peerj.15501 (PMC10259447; doi:10.7717/peerj.15501)
Supplement: Supplemental Information 1 [file peerj-11-15501-s001.docx]

**Supplementary File 1**

This file includes:

**Table S1. Relative Abundance of Bacterial Phyla in Three Focal Bee Species**

**Figure S1. Rarefaction plot displaying number of ASVs with 529 sequence reads per sample**

**Figure S2. Relative abundance of bacterial genera in each bee species.**

**Figure S3. Heatmap showing the relative abundance (0.1 to 1.0) of bacterial ASVs (rows) in individual bees (columns).**

**Supplementary Tables**

**Table S1. Relative Abundance of Bacterial Phyla in Three Focal Bee Species**

| Phyla | *A. mellifera* | *B. impatiens* | *B. griseocollis* |
| --- | --- | --- | --- |
| Actinobacteria | 0.24% | 1.17% | 1.56% |
| Bacteroidetes | 1.35% | 3.94% | 1.77% |
| Firmicutes | 20.68% | 23.44% | 13.57% |
| Proteobacteria | 77.73% | 71.46% | 83.10% |

**Supplementary Figures**


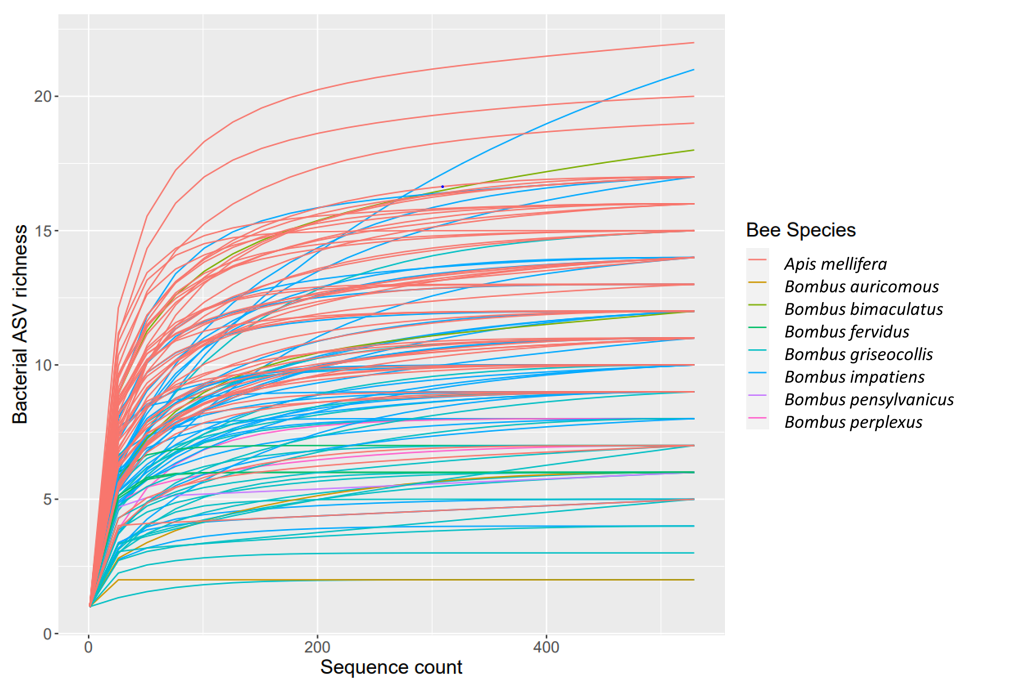
**Figure S1. Rarefaction plot displaying number of ASVs with 529 sequence reads per sample.**


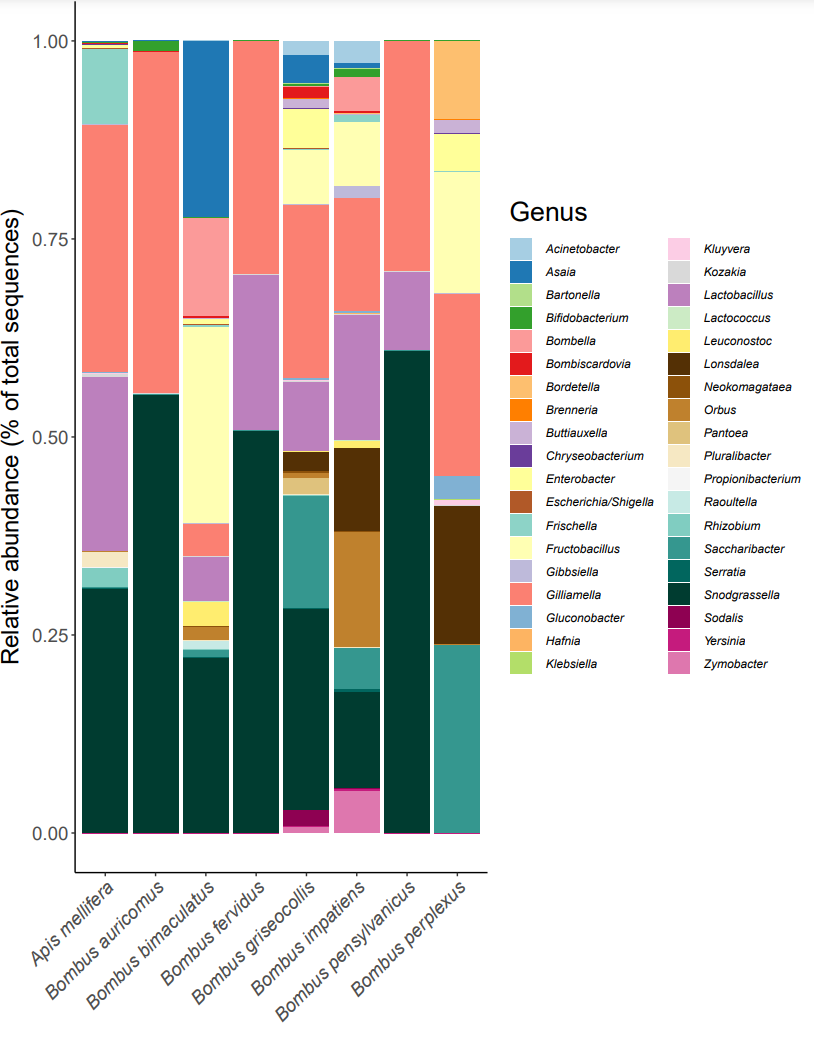


**Figure S2. Relative abundance of bacterial genera in each bee species.**

**
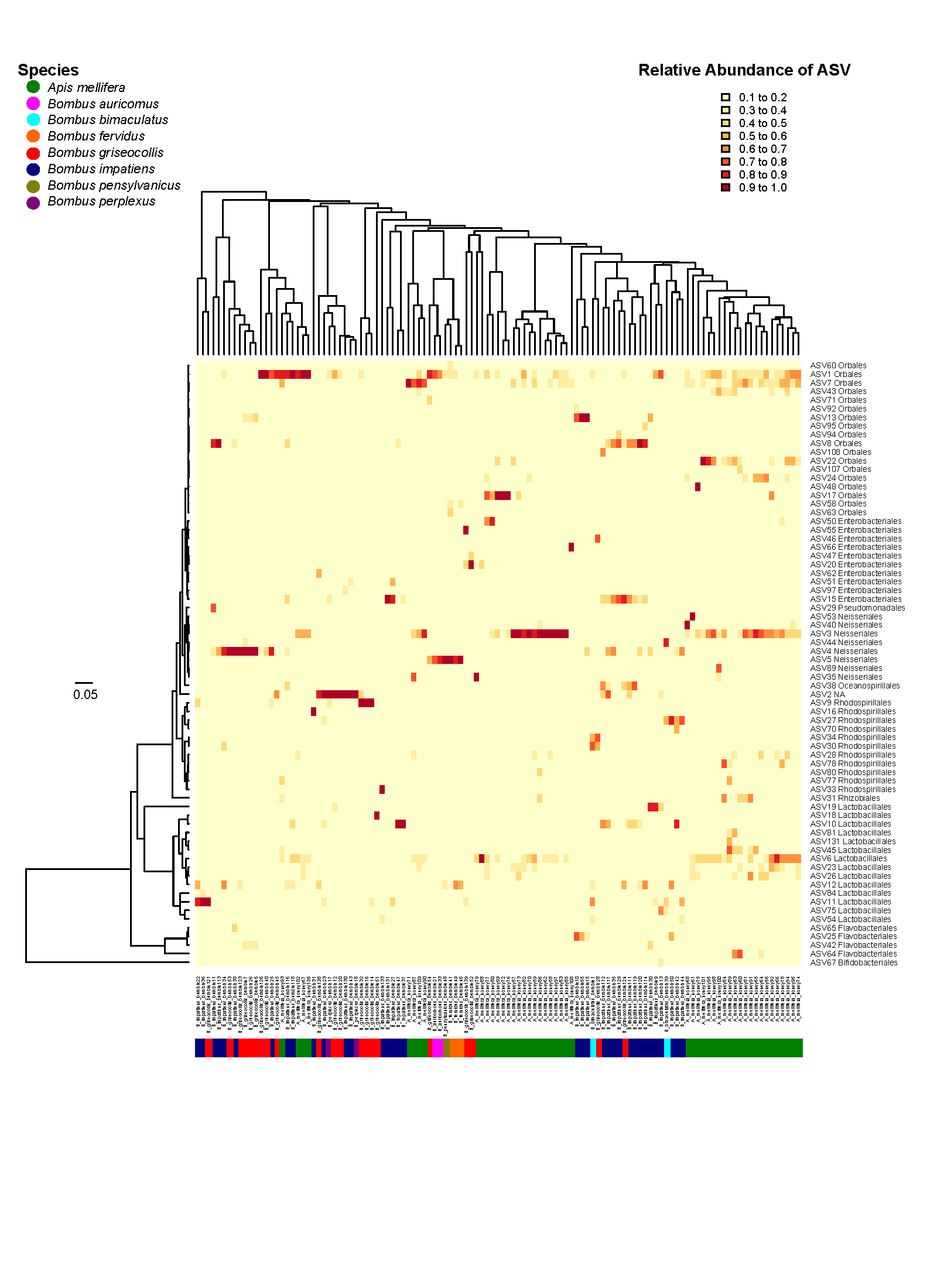
**

**Figure S3. Heatmap showing the relative abundance (0.1 to 1.0) of bacterial ASVs (rows) in individual bees (columns).** Columns are grouped based on the results of hierarchical clustering. Tree on the left of the heatmap indicates phylogenetic relationships between bacterial ASVs. Taxonomic orders of bacteria are shown on the right of the heatmap. Bacterial taxa common in focal species and mentioned in text are labeled by ASV numbers. Colors of heatmap indicate abundance classes of ASVs. *Apis mellifera* displays greater bacterial uniformity (e.g., ASV 3) compared to bumble bees. ASV host specificity can also be observed, with some having higher abundances (e.g., ASV 6) in one host species over another. Scale bar indicates the number of substitutions per nucleotide site along branches of the bacterial phylogenetic tree.
